# Retrospective biodosimetry using translocation frequency in a stable cell of occupationally exposed to ionizing radiation

**DOI:** 10.1093/jrr/rrv028

**Published:** 2015-04-28

**Authors:** Min Su Cho, Jin Kyung Lee, Keum Seok Bae, Eun-Ae Han, Seong Jae Jang, Wi-Ho Ha, Seung-Sook Lee, Joan Francesc Barquinero, Wan Tae Kim

**Affiliations:** 1 Department of Emergency Medical Preparedness, National Radiation Emergency Medical Center, Korea Institute of Radiological and Medical Sciences, 75 Nowon-gil, Nowon-gu, Seoul, South Korea; 2 Department of Surgery, Yonsei University Wonju College of Medicine, 162 Ilsan-dong, Wonju 220–701, Gangwon-do, South Korea; 3 Department of Biological Dosimetry, National Radiation Emergency Medical Center, Korea Institute of Radiological and Medical Sciences, 75 Nowon-gil, Nowon-gu, Seoul, South Korea; 4 Department of Health Physics, National Radiation Emergency Medical Center, Korea Institute of Radiological and Medical Sciences, 75 Nowon-gil, Nowon-gu, Seoul, South Korea; 5 Department of Animal Biology, Plant Biology and Ecology, Autonomous University of Barcelona, 08193 Bellaterra, Catalonia, Spain; 6 Division of Radiation Regulation, Korea Institute of Nuclear Safety, 62 Gwahak-ro, Yuseong-gu, Daejeon 305–338, South Korea

**Keywords:** translocation, retrospective biodosimetry, stable cells, occupational exposure

## Abstract

Two cases of hematological malignancies were reported in an industrial radiography company over a year, which were reasonably suspected of being consequences of prolonged exposure to ionizing radiation because of the higher incidence than expected in the general population. We analyzed chromosomal aberrations in the peripheral blood lymphocytes from the other workers who had been working under similar circumstances as the patients in the company. Among the subjects tested, 10 workers who belonged to the highest band were followed up periodically for 1.5 years since the first analysis. The aim of this study was to clarify pertinence of translocation analysis to an industrial set-up where chronic exposure was commonly expected. To be a useful tool for a retrospective biodosimetry, the aberrations need to be persistent for a decade or longer. Therefore we calculated the decline rates and half-lives of frequency for both a reciprocal translocation and a dicentric chromosome and compared them. In this study, while the frequency of reciprocal translocations was maintained at the initial level, dicentric chromosomes were decreased to 46.9% (31.0–76.5) of the initial frequency over the follow-up period. Our results support the long-term stability of reciprocal translocation through the cell cycle and validate the usefulness of translocation analysis as a retrospective biodosimetry for cases of occupational exposure.

## INTRODUCTION

Although ionizing radiation has been one of the most well known carcinogens in our environment [1], it is still challenging to demonstrate the association between previous exposure to ionizing radiation and cancer development. Two cases of myelodysplastic syndrome have recently been reported as a consequence of occupational exposure to ^192^Ir and/or ^60^Co in an industrial radiography company in Korea in 2011 [2]. The company, which had a total of 32 workers, in fact had one further case of myelodysplastic syndrome before the two cases had been reported; this additional case has only been briefly mentioned but was not presented in full in the report [2]. None of the previous records have indicated that any of the workers in the company exceeded the legal dose limit according to their thermoluminescent dosimeter (TLD) badges. Considering that the incidence of myelodysplastic syndrome in the Korean population in 2011 was 1.2 cases per 100 000 individuals [3], we had grounds for suspicion that the protracted exposure of the workers at the company played a role. In order to estimate the absorbed doses, we used biodosimetry techniques; in addition, we evaluated the clinical utility of the translocation assay as a retrospective biodosimetry technique. We analyzed chromosomal aberrations in the peripheral blood lymphocytes from all the other workers for initial analysis and followed up the 10 workers whose absorbed doses were in the highest band periodically thereafter.

**Fig. 1.**
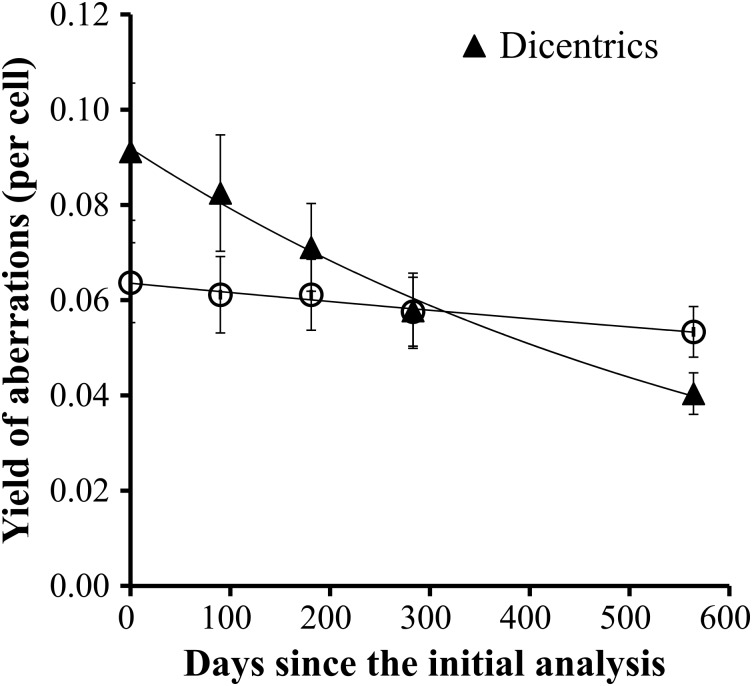
Time-course of the chromosome aberrations in the 10 follow-up workers at different periods since the initial analysis. The representative values of aberration yields were the mean values of dicentrics (black triangles) or translocations (white circles) per cell in the 10 workers at each time-point. Bars are standard errors.

Scoring dicentric chromosomes in solid-stained metaphase cells has been considered the most reliable method for estimating individual whole-body dose. However, this method has application limited to a certain scenario, where the exposure has been acute, largely uniform and recent [4]. In cases of occupational exposure, the scenario is more likely to be repetitive exposure to a lower dose over several years and not a single incident. Under circumstances where the exposure is repetitive or over several years, analyzing dicentric chromosomes may not be the most appropriate method. The consensus in the scientific community is that since a cell with dicentric chromosomes cannot survive through a cell division, scoring the dicentric chromosomes after a certain period can lead to underestimation of the dose.

In the case of prolonged exposure, a stable aberration such as reciprocal translocation is recommended as the biomarker of choice [4–6]. Recently developed techniques of fluorescence *in situ* hybridization (FISH) allow translocations to be detected more easily than microscopic observation with solid Giemsa staining or G-banding. Unlike the dicentrics assay, however, the main technical difficulty of the translocation assay is the lack of standardization between laboratories.

Theoretically, radiation-induced translocation should persist over time after irradiation, because the translocation does not cause an increase or decrease in genetic material, and therefore does not cause cell death through division. However, despite this theoretical assumption, many studies have shown that translocation frequencies decline over time in studies of accidentally exposed individuals or individuals exposed to radiation for therapeutic purposes [5–15]. The apparent controversy over the kinetics of chromosomal translocations as demonstrated in different studies may be a consequence of the differences between the assay technologies that have been used. From a technical point of view, the decline in translocation frequency can be explained by the co-occurrence of dicentrics and translocations in the same cell.

There is currently a scarcity of data that demonstrates the decline rate of reciprocal translocation when stable cells only are considered for the analysis. The aim of this study is to investigate the decline rates of reciprocal translocations in a stable cell compared with that of dicentrics. This shold help to validate the availability of translocation analysis as a retrospective biodosimetry for cases of occupational exposure.

## MATERIALS AND METHODS

### Study population

Two cases of hematological malignancies were reported over a one-year period, and were thought to be a consequence of occupational exposure to ^192^Ir and/or ^60^Co in an industrial radiography company in Korea in 2011 [2]. The reported cases of leukemia are briefly summarized in Table 1. For initial analysis, blood samples were collected from the other 32 workers in the same company (who had been working under similar circumstances to those of the reported cases) upon request by the MEST (Ministry of Education, Science and Technology) of Korea. The demographic characteristics of the 32 workers are described in Table 2. Among the subjects, 10 workers whose absorbed doses were at a level of 500 mGy or over were selected and followed up every 3 or 10 months for 1.5 years since the initial analysis.

**Table 1.** Summary of the cases of hematological malignancies caused by radiation exposure in industrial radiography workers that were reported in Korea in 2011

	Case 1	Case 2
Sex	M	M
Age (years) at Dx	35	26
Work duration (years)^a^	10.0	2.5
BM finding	AML with myelodysplasia-related changes	Refractory anemia with excess blasts
Cytogenetic findings	44,XY,t(3;?)(p13;?),-7, add(17)(p11.2), -18,∼2dmin[20]	44,XY,-5,-7, add(16)(q12.1)[17] /46,XY[3]
Doses (mGy)		
by DIC analysis	1200	1046
by 1,2,4-FISH	3601	1713
Outcome	Dead	Dead

AML = acute myeloid leukemia, BM = bone marrow, Dx = diagnosis, FISH = fluorescence *in situ* hybridization, M = male. ^a^Duration from the initial work of radiography to diagnosis of the disease.

**Table 2.** Demographic characteristics of the 32 workers who were enrolled in initial analysis

Case	Sex	Age (years)	Smoking Hx (pack-year)	Work duration (years)
1	M	35	10	5.0
2	M	33	10	2.8
3	M	36	16	3.3
4	M	27	7	2.8
5	M	39	20	5.7
6	M	34	10	5.0
7	M	30	9	3.5
8	M	35	3	5.6
9	M	39	10	8.6
10	M	30	5	2.2
11	M	30	10	3.7
12	M	29	12	3.3
13	M	33	15	9.8
14	M	31	10	2.0
15	M	35	15	7.8
16	M	58	30	2.3
17	M	40	18	8.3
18	M	28	5	1.1
19	M	43	25	6.1
20	M	38	17	8.6
21	M	36	15	9.3
22	M	37	18	9.4
23	M	39	5	18.2
24	M	36	Never	10.0
25	M	43	16	14.2
26	M	53	5	11.0
27	M	24	Never	0.9
28	M	37	15	14.3
29	M	32	10	2.3
30	M	30	5	2.0
31	M	27	5	0.6
32	F	26	Never	0.0^a^
Mean		35.1	12.1	5.9
SD		7.2	6.4	4.5

M = male, F = female, Hx = history. ^a^office work only.

All of the enrolled subjects in the study provided written consent for the blood sampling procedure and the use of their information in this study. The Institutional Review Board of the Korea Institute of Radiological and Medical Sciences approved the study design, including the involvement of human subjects (K-1301-002-033).

### Analysis of dicentric chromosomes by solid Giemsa staining

Heparinized whole blood samples were processed to be cultured within 24 h after collection. The process of culturing, harvesting, staining and scoring was performed according to our technical specifications [16], which are based on the IAEA recommendations [17]. Briefly, 1 ml of heparinized whole blood was cultured for 48 h in 9 ml of RPMI-1640 medium supplemented with 20% (v/v) of fetal calf serum, 2% phytohemagglutinin, and antibiotics. Colcemid at a concentration of 0.07 µg/ml was added 24 h before harvesting. After hypotonic treatment with 0.075 M KCl, the cells were fixed in a 3:1 solution of methanol-acetic acid. The cells were spread onto a slide and stained with 4% Giemsa solution in phosphate buffer for 10 min. Metaphase cells were scanned automatically using the Metafer 4 platform (MetaSystems GmbH, Altlussheim, Germany), and 1000 cells were selected for scoring. To calculate the absorbed dose, the yield of dicentrics with acentric fragments was extrapolated from the dose–response curve, which had already been established in our laboratory [16, 17].

### Analysis of translocation by fluorescence *in situ* hybridization

Heparinized whole blood samples were processed to be cultured within 24 h after collection. The process of preparing metaphase cells on slides was the same as the dicentrics assay. Metaphase cells on slides were prepared with 2X SSC for 2 min at 72°C and dehydrated with a series of ethanol solutions (70, 95 and 100%) for 1 min at each concentration. To hybridize the FITC-labeled probes (MetaSystems GmbH, Altlussheim, Germany) to chromosomes 1, 2 and 4, the probe mixture and target sequences were co-denatured at 75°C for 2 min and incubated at 37°C for 72 h. After rinsing with 0.4X SCC at 72°C and 2X SSC at room temperature for 30 s, the cells were counterstained with 4′,6′-diamidino-2-phenylindole (DAPI). Metaphase cells were scanned automatically using the Metafer 4 fluorescence scanning system (MetaSystems GmbH, Altlussheim, Germany).

To extrapolate the translocation frequency to the dose–response curve, we scored two-way apparently simple translocations, [t(Ab)t(Ba)], and the one-way form, t(Ba) and/or t(Ab) only in stable cells. Translocations in the cells that harbor unstable aberrations, such as dicentrics, rings or acentric fragments affecting one or both of the painted and unpainted chromosomes, were excluded.

### Dose estimations

The absorbed dose for each of individuals tested was calculated from the measured yield of dicentrics and translocations by fitting to dose–response calibration curves that we had previously constructed [16]. In brief, for the calibration curve, ^60^Co was used as a source at a dose rate of 0.5 Gy/min. A linear–quadratic curve containing 10 dose points (0, 0.1, 0.25, 0.5, 0.75, 1, 2, 3, 4 and 5 Gy) was constructed with 95% confidence intervals, based on the data of yield and distribution of dicentrics and translocations for each radiation dose. The equation for dicentrics is *Y* = *0.00146* + *(0.02688) D* + *(0.07171) D^2^*. The equation for translocations is *Y* = *0.00240* + *(0.01124) D* + *(0.01752) D^2^*.

## Statistical methods

The cell-to-cell distribution of the chromosomal aberrations was verified to see if it followed a Poisson distribution by using the dispersion index, *D* = σ^2^/*Y*, and the u value, which represents a normalized unit of the index [17].

Formulas of the decay rate and the half-life of chromosomal aberrations shown in equations (1) and (2) were fitted to the data for each subject:

(1) $$Y(t)=Y(0){e^{-}}^{\lambda t},$$

(2) $$T_{1/2}=ln(2)/\lambda ,$$

where *Y(t)* is the yield of aberrations, *Y(0)* is the initial yield and λ is the time constant for decay.

## RESULTS

### Initial biodosimetry for the 32 workers

The mean frequencies of the dicentric chromosomes and translocations in the 32 radiography workers were 0.045 per cell and 0.031 per cell, respectively, which were significantly higher than the background level, based on our previous study [16]. The mean absorbed doses for subjects whose frequencies of chromosomal aberrations were above the limit of detection were 588.1 mGy for recent exposure and 893.4 mGy for retrospective exposure (Table 3). Distribution of the aberrations among cells followed Poisson statistics; u values were in the range of ± 1.96 in most of the subjects. Although the 20 workers with doses, either recent or retrospective, of 500 mGy or over were included for follow-up analysis, only 10 were available for regular blood sampling thereafter. The results of the initial biodosimetry are summarized in Table 3.

**Table 3.** Initial biodosimetry results for the 32 workers

Case	Unstable aberrations			Stable aberrations		
	Yield of DICs per cell	u	Dose by DICs (mGy)	Yield of TR per cell	u	Dose by TR (mGy)
1	0.210	0.93	1528	0.115	2.58	2235
2	0.138	1.81	1205	0.059	0.22	1505
3	0.113	1.06	1074	0.090	3.24	1941
4	0.108	0.51	1046	0.073	−0.03	1713
5	0.105	1.52	1029	0.062	2.50	1544
6	0.081	1.53	882	0.088	0.60	1913
7	0.079	−0.05	869	0.052	1.74	1392
8	0.073	6.4	829	0.063	2.18	1567
9	0.070	−0.27	808	0.040	−0.82	1175
10	0.066	−0.10	780	0.027	1.10	907
11	0.064	−0.72	765	0.036	−0.70	1107
12	0.061	0.13	743	0.034	2.27	1060
13	0.056	2.79	705	0.057	−0.43	1471
14	0.035	0.53	522	0.013	−0.26	531
15	0.030	−0.66	471	0.024	−0.53	835
16	0.022	−0.48	380	0.032	3.56	1018
17	0.020	−0.44	354	0.014	3.01	554
18	0.015	−0.32	286	0.016	5.43	617
19	0.012	−0.26	239	0.027	−0.59	907
20	0.011	−0.23	223	0.005	−0.10	180
21	0.011	−0.23	223	0.007	−0.14	284
22	0.010	−0.21	205	0.007	−0.14	284
23	0.008	−0.21	168	0.009	−0.19	372
24	0.008	−0.17	168	0.005	−0.10	180
25	0.007	−0.14	148	0.013	−0.28	521
26	0.006	−0.12	126	0.005	9.90	180
27	0.005	−0.10	103	0.003	0.0	<100^a^
28	0.004	−0.08	<100^a^	0.008	−0.17	329
29	0.004	−0.08	<100^a^	0.005	−0.10	180
30	0.004	−0.08	<100^a^	0.004	−0.08	120
31	0.003	0.0	<100^a^	0.005	−0.10	180
32	0.002	0.0	<100^a^	0.002	0.0	<100^a^
Mean	0.045		588.1	0.031		893.4

DICs = dicentric chromosomes, TR = translocations. ^a^The lowest level of the detectable absorbed dose in our laboratory is 100 mGy.

### Decline of chromosomal aberrations in follow-up biodosimetry for the 10 workers

Over a period of 564 days (1.5 years) since the initial analysis, four more tests were regularly performed at intervals of 3 or 10 months (Fig. 1). The follow-up results are summarized in Tables 4 and 5. The decay constant, l, gives rate of decrease of chromosome aberrations of each individual for the follow-up period. The yields of dicentrics declined to 49.6% (range 31.0–64.3%) of the initial level at the last follow-up (Table 4), and the calculated half-life was 554.4 ± 50.4 days (1.5 ± 0.1 years). On the contrary, the yields for the translocations showed only a small decrease or even a slight increase during the follow-up period, with the average final level standing at 97.8% (range 85.4–110.2%) of the initial level (Table 5). The expected half-life of reciprocal translocations in a stable cell was beyond the normal human life expectancy.

**Table 4.** Time-course of the frequencies of the dicentric chromosomes in the 10 follow-up workers at different periods after the initial analysis

Case	F/U (Days^a^)	1st (0)	2nd (90)	3rd (181)	4th (283)	5th (564)	λ (× 10^−3^)
1	Y_d_(/cell) D_d_(mGy)	0.210 1528	0.178	0.136	0.118	0.065	2.079
3	Y_d_(/cell) D_d_(mGy)	0.113 1074	0.100	0.090	0.062	0.044	1.672
5	Y_d_(/cell) D_d_(mGy)	0.105 1029	0.108	0.099	0.070	0.055	1.147
6	Y_d_(/cell) D_d_(mGy)	0.081 882	0.080	0.064	0.062	ND	0.945
7	Y_d_(/cell) D_d_(mGy)	0.079 869	0.067	0.064	0.052	0.037	1.345
8	Y_d_(/cell) D_d_(mGy)	0.073 829	0.060	0.053	0.033	0.031	1.519
9	Y_d_(/cell) D_d_(mGy)	0.070 808	0.065	0.058	0.045	0.045	0.783
11	Y_d_(/cell) D_d_(mGy)	0.064 765	0.064	0.060	0.062	0.031	1.285
12	Y_d_(/cell) D_d_(mGy)	0.061 743	0.056	0.049	0.044	0.031	1.200
13	Y_d_(/cell) D_d_(mGy)	0.056 705	0.047	0.038	0.030	0.025	1.442
Mean	Y_d_(/cell) D_d_(mGy)	0.091 923	0.083	0.071	0.058	0.040	

F/U = follow-ups, Y_d_ = yield of dicentrics, D_d_ = absorbed dose by dicentrics assay, λ = the decay constant, ND = not done. ^a^Days since the initial analysis.

**Table 5.** Time-course of the frequencies of translocations affecting painted chromosomes 1, 2 and 4 in the 10 follow-up workers at different periods after the initial analysis

Case	F/U (Days^a^)	1st (0)	2nd (90)	3rd (181)	4th (283)	5th (564)	λ (×10^−3^)
1	Y_t_(/cell) D_t_(mGy)	0.115 2235	0.111	0.107	0.101	ND	0.473
3	Y_t_(/cell) D_t_(mGy)	0.090 1941	0.075	0.072	0.071	0.077	0.281
5	Y_t_(/cell) D_t_(mGy)	0.062 1544	0.070	0.072	0.066	0.059	0.074
6	Y_t_(/cell) D_t_(mGy)	0.088 1931	0.083	0.081	0.080	ND	0.319
7	Y_t_(/cell) D_t_(mGy)	0.052 1392	0.044	0.039	0.042	0.055	−0.010
8	Y_t_(/cell) D_t_(mGy)	0.063 1567	0.072	0.068	0.066	0.068	−0.135
9	Y_t_(/cell) D_t_(mGy)	0.040 1175	0.034	0.048	0.037	0.040	−0.009
11	Y_t_(/cell) D_t_(mGy)	0.036 1107	0.043	0.038	0.038	0.040	−0.172
12	Y_t_(/cell) D_t_(mGy)	0.034 1060	0.030	0.029	0.029	0.033	0.053
13	Y_t_(/cell) D_t_(mGy)	0.057 1471	0.051	0.059	0.047	0.055	0.057
Mean	Y_t_(/cell) D_t_(mGy)	0.064 1542	0.061	0.061	0.058	0.053	

F/U = follow-ups, Y_t_ = yield of translocations, D_t_ = absorbed dose by tanslocation assay, λ = the decay constant, ND = not done. ^a^Days since the initial analysis.

## DISCUSSION

Recently two cases of industrial radiography workers who were diagnosed with leukemia after several years of work were reported in a peer-reviewed publication in Korea [2]. There was, in fact, one more case that was not included but was briefly mentioned in the report (Case 4 in Tables 2–3) in the same company. Although the level of TLD badges had never been reported to exceed the legal limit, the absorbed doses of several workers—5 by a dicentric analysis and 13 by translocation assay out of a total 32 workers—were above 1.0 Gy (Table 3). An interesting finding is that the both cases in the report showed monosomy 7 as a somatic aberration in the bone marrow. Monosomy 7 or 7q deletion is a frequently reported chromosomal aberration in therapy-related myelodysplastic syndrome [18, 19]. An *in vitro* evidence of higher incidence of chromosomal aberration on chromosome 7 has also been reported in a long-term cell culture after irradiation [20]. This cytogenetic finding, however, could not demonstrate the causal relationship between the radiation work and the disease in the case. The cases were legally acknowledged as a consequence of prolonged and repetitive exposure to an ionizing radiation based on the significantly higher incidence of leukemia among the company workers compared with the general population and the results of biological dosimetry performed in this study.

The fact that the recorded doses of personal dosimeters (TLD badges) have never exceeded the legal limit is another issue to be discussed. According to information gained from an interview with workers, they did not wear their dosimeter badges all the time. The absorbed doses estimated by cytogenetic biodosimetry revealed that the whole-body doses were markedly increased, for example, Case 1 showed >2 Gy of whole-body dose by the FISH technique using painting probes (Table 3). Taking this into account, this study was an example case which translocation frequency was supplementarily used to assess the exposed dose for long-term radiation workers who frequently did not wear TLD badges.

The frequency of chromosomal translocation in the peripheral blood lymphocytes is currently the most reliable biomarker used for retrospective biodosimetry. Long-time persistence of translocation frequency after irradiation has sound scientific support, and technical advances in the various kinds of FISH techniques provide technical feasibility for using the translocation analysis as a gold standard technique. However, there have been some controversies regarding the persistence of translocations. Several studies on time-course of translocation frequencies in radiation accidents have reported varying rates of decline. For instance, no substantial decrease in the translocation frequencies was reported in employees exposed in the Chernobyl accident from 5 to 8 years [21]. On the contrary, the study of the victims from the Estonia accident showed that the frequencies of translocations remained constant only for 2 years and decreased thereafter [22]. Many papers have reported that the translocation frequencies increase with aging in normal populations [23–25], but the aging effect was hard to determine in accidental cases. The frequency of the dicentric chromosome, on the other hand, has consistently been reported to decrease rapidly after the initial exposure. In the same study of the Estonia accident, a rapid decline in dicentric frequency was noted during the first year [22]. A study of three victims from the serious Tokai-mura accident reported a 13.5-months half-life of dicentric chromosomes [26]. Our results were also consistent with previous studies regarding the half-life of dicentric frequency, which was determined to be 1.5 years.

The various time-courses of translocation frequencies, as described above, were considered as unavoidable complications when we assessed the initial doses of previously exposed victims [11]. In a recent study, Rodriguez *et al.* showed that the persistence of translocations depends on several factors, such as the initial exposure dose, scenario of exposure (such as partial or whole-body exposure), type of translocation enumerated, and the criteria of analysis used in the construction of the calibration curves [27]. We considered the type of translocations that were counted, and co-harboring of stable and unstable aberrations in the same cell are the two main factors that cause discrepancy in the decline rate. Therefore, in order to avoid these potentially confounding factors in our study, we counted only two-way reciprocal translocations that were transcribed into [t(Ab)t(Ba)] and one-way apparently simple translocations, such as t(Ab) and/or t(Ba) in stable cells. To consider only stable cells, we excluded any translocations observed in the cells that also harbored unstable aberrations, such as dicentrics, rings, or acentric fragments affecting the painted and/or unpainted chromosomes. These enumeration criteria were also applied when we generated the dose–response curve from which the translocation frequencies of follow-up workers were extrapolated.

The results of this study should be interpreted in the context of recognizing several limitations. First, we could not confirm the exposure episode of the 32 subjects. Selection of the subjects was entirely dependent on the possibility of exposure causing a higher incidence of hematologic malignancies among the company workers. However, the three cases of leukemia or myelodysplastic syndrome (two were officially reported and one was only mentioned in the local journal) were subsequently admitted as work-related diseases by the relevant legal agencies in Korea. Therefore, it was deemed reasonable to enroll the other workers for initial analysis and select those with the highest dose band for follow-up. Second, we do not have the baseline levels of chromosome aberrations for each subject before they began their jobs at the company. Due to the lack of baseline levels for each worker, we could not determine the exposure of workers who belonged to the lower dose band in the initial analysis. Finally, the period of follow-up was not sufficiently long to observe the initial dicentric frequency decline to the background level. Several visits for blood drawing over a longer period may not be desirable or convenient for the enrolled workers. Instead, we calculated the half-lives of lymphocytes with chromosome aberrations, both dicentric chromosomes and translocations.

There are few data on the *in vivo* half-life of chromosomal aberrations, because it is difficult to follow up the blood samples of victims of radiation exposure [28–29]. Despite the limitations, this study has provided valuable data regarding the *in vivo* half-life of dicentric chromosomes and shown the persistence of stable chromosomal aberrations enumerated in stable cells. Based on these data, we have demonstrated the usefulness of the translocation assay involving painting of FISH probes for retrospective biodosimetry for victims of prolonged and repetitive exposure.

## FUNDING

This work was supported by the Nuclear Safety Research Program through the Korea Radiation Safety Foundation, granted financial resources from the Nuclear Safety and Security Commission of Korea (No. 1203024) and R&D Program granted by the Ministry of Education, Science and Technology of Korea (No. 50445-2014). Funding to pay the Open Access publication charges for this article was provided by the Nuclear Safety Research Program of the Nuclear Safety and Security Commission of Korea (No. 1203024).
